# Elevated Chinese visceral adiposity index increases the risk of stroke in Chinese patients with metabolic syndrome

**DOI:** 10.3389/fendo.2023.1218905

**Published:** 2023-06-29

**Authors:** Zeyu Liu, Qin Huang, Bi Deng, Minping Wei, Xianjing Feng, Fang Yu, Jie Feng, Yang Du, Jian Xia

**Affiliations:** ^1^ Department of Neurology, Xiangya Hospital, Central South University, Changsha, Hunan, China; ^2^ Clinical Research Center for Cerebrovascular Disease of Hunan Province, Central South University, Changsha, Hunan, China; ^3^ National Clinical Research Center for Geriatric Disorders, Xiangya Hospital, Central South University, Changsha, Hunan, China

**Keywords:** Chinese visceral adiposity index, stroke, metabolic syndrome, obesity, visceral fat tissue

## Abstract

**Introduction:**

Patients with Metabolic Syndrome (MetS) are considered at high-risk for incident stroke. An indicator of visceral adiposity dysfunction, the Chinese Visceral Adiposity Index (CVAI) is used to evaluate the dysfunction of visceral fat. Given the impact of visceral adiposity dysfunction on elevating cardiovascular hazards, this study aimed to examine the association between CVAI and stroke risk in MetS patients.

**Method:**

Between November 2017 and December 2018, a total of 18,974 individuals aged ≥40 underwent standardized in-person clinical interviews in Hunan Province, with 6,732 meeting the criteria for MetS. After the baseline survey was completed, subsequent surveys were conducted biennially. The study was split into two stages performed at baseline and after two years. During the former, receiver-operating characteristic curves were used to assess the accuracy of using baseline CVAI in diagnosing MetS. After two years, we examined the association between CVAI and incident stroke in MetS patients using logistic regression, subgroup analysis, and restricted cubic spline (RCS) analysis.

**Result:**

As evidenced by a higher AUC (AUC:0.741), CVAI demonstrated superior diagnostic performance relative to body mass index (AUC:0.631) and waist circumference (AUC:0.627) in diagnosing MetS. After a 2-year follow-up, 72 MetS patients had a stroke event. There was a robust positive correlation between incident stroke and CVAI in patients with MetS. Each 1 SD increase in CVAI was associated with a 1.52-fold higher risk of stroke after adjustment for confounding factors (aOR=1.52, 95%CI: 1.18-1.95). The RCS demonstrated a reduced risk of stroke for MetS patients when the CVAI was below 110.91. However, no significant correlation was detected between CVAI and stroke in non-MetS patients.

**Conclusion:**

Our findings recommend CVAI as a superior screening tool for detecting MetS and suggest that reducing CVAI can mitigate the risk of stroke in patients with MetS.

## 1 Introduction

Stroke ranks third in terms of morbidity and second in terms of disability-adjusted life-years worldwide (1, 2), resulting in an economic burden greater than 721 billion USD, which is equivalent to 0.66% of the global gross national product (3).

Due to an aging population, the stroke burden in China is escalating. In 2020, a nationwide survey of 676,394 adults aged ≥40 years revealed an estimated incidence and mortality rate for stroke of 502.2 per 100,000 person-years and 343.4 per 100,000 person-years, respectively (4). It is therefore important to identify effective primary prevention and early intervention strategies for stroke (5).

Metabolic syndrome (MetS) is an umbrella term that refers to multiple metabolic abnormalities, including hypertension, central obesity, impaired glucose regulation, and atherogenic dyslipidemia (6). The improvement in the general standard of living has accompanied a significant rise in the incidence of MetS (7), as well as a concomitant elevation in the risk of stroke (8–10).Identifying risk factors for incident stroke in patients with MetS would help to mitigate the anticipated rise in stroke burden resulting from the increased morbidity of MetS. As such, it is essential to identify the risk factors of incident stroke for MetS patients.

Adipose tissue is important not only for storing energy but also regulating endocrine function through the secretion of adipokines (11). Adipose tissue is categorized as either visceral fat tissue (VAT) or subcutaneous fat tissue (SAT) according to its location. The accumulation of VAT is strongly associated with increased cardiometabolic risk (12–15). The study conducted by Huang et al. demonstrated significant correlations between VAT mass and a wide range of CVD outcomes, including but not limited to coronary heart disease, cardiac arrhythmia, vascular diseases, and stroke (16). Additionally, another Mendelian Randomization Study furnished proof of a substantial causal link between VAT and ischemic stroke, as opposed to intracerebral hemorrhage (17). In general, visceral obesity is among the most evident clinical features of MetS (18). Obese patients with MetS have a higher risk of developing cardiovascular disease (CVD) than non-obese patients (19). However, whether a higher degree of visceral adiposity in MetS patients is associated with an increased risk of incident stroke remains poorly characterized.

At present, body mass index (BMI) and waist circumference (WC) are the most common methods for estimating adiposity and assessing central obesity in patients with MetS. However, these metrics are limited: BMI cannot distinguish between the accumulation of fat-free mass and fat, leading to the misdiagnosis of muscular individuals as overweight or obese (20); WC is marred by poor reliability and is inadequate for differentiating between subcutaneous and visceral fat (21). While more technologically advanced alternatives, such as magnetic resonance imaging and computed tomography, are considered gold standards, their technical complexity and high cost prohibit their use in routine clinical practice (22). The need for a reliable and low-cost indicator of visceral adiposity has prompted the development of novel indices based on combining anthropometric and biochemical assessments: e.g., the visceral adiposity index (VAI) and Chinese visceral adiposity index (CVAI) (23). In 2016, a CVAI was established that utilizes clinically available metabolic parameters, including age, BMI, WC, triglycerides (TG), and high-density lipoprotein cholesterol (HDL-C) (24). CVAI is highly correlated with visceral fat area and outperforms BMI, WC, or VAI in the diagnosis of diabetes and hypertension among Chinese population (25, 26). Furthermore, elevated CVAI is significantly associated with increased risks of carotid plaque and CVD (27–29). Few studies, however, have investigated an association between CVAI and incident stroke in patients with MetS. To resolve this dearth in the literature, the present study investigates the relationship between CVAI and incident stroke in a Chinese population.

## 2 Materials and methods

### 2.1 Study design and population

The present study used patient data collected by the China Stroke High-risk Population Screening and Intervention Program (CSHPSIP): an ongoing population-based screening project conducted by the China Stroke Prevention Project Committee (30). The program aims to mitigate stroke risk by addressing the prevalence of stroke risk factors through screening, physical examination, and comprehensive interventions. The CSHPSIP enrolled community-dwelling adults who were (1) aged >40 years, (2) resided in the community for >6 months, and (3) provided informed consent (4). The protocol for the program were reviewed and approved by the Institutional Review Board at the Capital Medical University Xuanwu Hospital (No. 2012045).

The present study used data obtained from individuals residing in Hunan Province, a region featuring a relatively elevated incidence of stroke within China (4). 54338 individuals enrolled from twenty-six communities (thirteen urban areas and thirteen rural areas) in the province were administered a baseline survey between January 2017 and December 2018. All eligible participants underwent standardized in-person clinical interviews. After the baseline survey was completed, subsequent surveys were conducted biennially.

A total of 20,487 respondents participated in the follow-up survey two years after completing the baseline survey. Individuals with incomplete sociodemographic information, missing anthropometric measures, or lacking laboratory assay results due to unsuccessful blood collection were excluded from the study.

Of the 18,974 participants included in the final analysis, 6,732 were diagnosed with MetS according to the Chinese Guidelines for the Prevention and Treatment of Type 2 Diabetes (2020 edition). The criteria for defining the detailed components of MetS were as follows (31): (1) abdominal obesity, ascertained by a WC of ≥ 90 cm in males or ≥ 85 cm in females; (2) hypertension, defined as a blood pressure of ≥ 130/85 mmHg or a history of hypertension; (3) hyperglycemia, indicated by a fasting plasma glucose (FBG) level of ≥ 6.10 mmol/L, a 2-h plasma glucose level of ≥7.8 mmol/L, or a diagnosis of type 2 diabetes mellitus; (4) high TG, determined by a fasting TG level of ≥1.70 mmol/L; and (5) low HDL-C, indicated by an HDL-C level of ≤ 1.04 mmol/L. Patients who fulfilled any three of the aforementioned five criteria were diagnosed with MetS.

In parallel with the survey schedule, the study was split into two stages performed at baseline and after two years (**Supplemental Figure 1**). During the former, we examined the associations between baseline CVAI levels and MetS diagnosis, as well as assessed the diagnostic efficacy of CVAI in detecting MetS. After two years, we investigated the relationship between CVAI and incident stroke risk among patients diagnosed with MetS.

### 2.2 Baseline data collection and anthropometry measurements

Data on medical, socio-demographic, anthropometric, and lifestyle-related variables were obtained by trained interviewers or medical staff. Demographic information, including age, sex, education level, economic status, lifestyle risk factors (tobacco use, alcohol consumption, and physical activity), medical history (hypertension, diabetes mellitus, dyslipidemia, stroke, and atrial fibrillation), and family medical history of stroke was collected. Education level was classified as “primary school or below,” “middle school,” and “high school or above.” Income was stratified as “<5000 Chinese Yuan (CNY),” “5000–9999 CNY,” “10000–19999 CNY,” and “≥20,000 CNY.” The definition of alcohol consumption in this study was the regular intake of alcoholic beverages at a frequency of three or more times per week, with a minimum of 100 mL per drinking episode. Smoking was defined as the act of smoking continuously or cumulatively for a period exceeding six months. Physical inactivity refers to the absence of moderate-to-vigorous physical activity for >150 minutes/week or vigorous-intensity physical activity for >75 minutes/week (4). Diabetes was defined as a fasting plasma glucose level of ≥ 7.0 mmol/L (126 mg/dL), a previous diagnosis of diabetes mellitus, or the use of antidiabetic medication or insulin (32). Hypertension was defined as a blood pressure of ≥140/90 mmHg, a history of hypertension, or the use of antihypertensive medication (33). Dyslipidemia was defined as serum total cholesterol (TC) concentration ≥ 6.22 mmol/L (240 mg/dL), and/or low-density lipoprotein cholesterol (LDL-C) concentration ≥ 4.14 mmol/L (160 mg/dL), and/or TG concentration ≥ 2.26 mmol/L (200 mg/dL), and/or HDL-C concentration <1.04 mmol/L (40 mg/dL), or previous history of hyperlipidemia and currently taking lipid-lowering drugs (34). Atrial fibrillation was defined as electrocardiographic evidence of atrial fibrillation or treatment for atrial fibrillation.

Each participant underwent a physical examination conducted by a qualified nurse or physician. Height and weight were measured to a precision of 0.1 cm and 0.1 kg, respectively. BMI was calculated by dividing body mass (kilograms) by the height (meters) squared. The WC was measured to a precision of 0.1 cm at the highest point of the iliac crest during minimal respiration. In accordance with previous studies, general obesity was defined as a BMI of ≥25 kg/m^2^ following Asian-specific criteria (35); abdominal obesity as a WC of ≥90 cm for men and a WC of ≥85 cm for women (31). Blood pressure was measured twice by an examining nurse or physician at an interval of 15 min. The average between the two measurements was used as the final datum.

### 2.3 Biochemical measurements

Blood samples were collected after an 8-hour fast and analyzed using the HP-AFS/3 automatic immunoassay system A3 Specific Protein Analyzer with supporting reagents (Shijiazhuang Hebo Biotechnology Co., Ltd., Shijiazhuang, China) on the same day of collection. The biochemical indicators assessed included fasting blood glucose (FBG), Hemoglobin A1c (HbA1c), TC, TG, LDL-C, and HDL-C.

### 2.4 Definition of CVAI

CVAI scores were computed using sex-specific formulas as follows (36):

Males: CVAI = −267.93 + 0.68 × age (years) + 0.03 × BMI (kg/m^2^) + 4.00 × WC (cm) + 22.00 × log10(TG [mmol/L]) − 16.32 × HDL-C (mmol/L);

Females: CVAI= −187.32 + 1.71 × age (years) + 4.32 × BMI (kg/m^2^) + 1.12 × WC (cm) + 39.76 × log10(TG [mmol/L]) − 11.66 × HDL-C (mmol/L).

### 2.5 Definition of outcome incident stroke

All incidents of stroke, including ischemic stroke, intracerebral hemorrhage, and subarachnoid hemorrhage, were documented during the survey period. The diagnosis of stroke was confirmed either through neurological imaging (brain computed tomography or magnetic resonance imaging) or a diagnosis certificate from a secondary or higher medical unit. However, due to limitations in data collection methods, we were unable to record the exact onset time of stroke.

### 2.6 Statistical analysis

All continuous variables were non-normally distributed and are presented as medians with interquartile ranges (IQR). Categorical variables are presented as percentages. The baseline characteristics of participants without MetS were compared to those of patients with MetS using the Mann-Whitney test for continuous variables and the Chi-square test for categorical variables. The baseline characteristics of MetS patients with and without stroke were analyzed in the same manner. Furthermore, to demonstrate the baseline characteristics of CVAI, we stratified patients into quartiles based on their initial CVAI levels. Differences in baseline variables between groups were assessed using either the Jonckheere-Terpstra trend test or the Chi-square test for trends.

Multivariate logistic regression models were used to assess the correlation between CAVI and MetS diagnosis. Various multivariable models with different levels of adjustment were employed. Model 1 incorporated individual characteristics, such as age, sex, education level, and economic status. Model 2 further included lifestyle risk factors, like smoking, alcohol consumption, and physical activity; medical history of hypertension, diabetes mellitus, dyslipidemia, and stroke; and family medical history of stroke. The variables included in the models all met the criteria of tolerance > 0.1 and variance inflation factor < 10. The diagnostic performance of CVAI in detecting MetS was compared with that of BMI and WC using receiver operating characteristic (ROC) curve analyses. In addition, we employed a restricted cubic spline to evaluate the dose-response relationship between CVAI and MetS (knots on the 5th, 25th, 75th, and 95th percentiles).

After a 2-year follow-up, multivariate logistic regression models were used to evaluate the correlation between stroke risk in MetS patients and CVAI, as well as other adiposity measures. The multivariable models were consistent with those described above. Model 1 contained individual characteristics (age, sex, education, economic status). Model 2 added lifestyle risk factors and medical history (smoking, alcohol drinking and physical activity, hypertension, diabetes mellitus, dyslipidemia, atrial fibrillation, prior stroke, family medical history of stroke on the base of model 1. The odds ratio (OR) was calculated with a 95% confidence interval (CI) for the presence of incident stroke. The dose-response relationship between CVAI or other adiposity measures and stroke risk was evaluated with a restricted cubic spline. Subgroup analyses were further performed to investigate the relationship between CVAI and stroke in various subgroups based on age (≥60, <60 years), sex (male, female), diabetes (yes, no), hypertension (yes, no), dyslipidemia (yes, no), prior stroke (yes, no), current smoking status (yes, no), current drinking habits (yes, no), and physical activity level (lacking or not lacking exercise). The p-value for an interaction between a subgroup variable and CVAI was assessed in each subgroup analysis.

SPSS version 25.0 (IBM SPSS, Armonk, NY, USA) and R version 4.2.3 (R Development Core Team, Vienna, Austria) were used for all statistical analyses. A two-tailed P-value of <0.05 was considered to indicate statistical significance.

## 3 Results

### 3.1 Characteristics of the study participants

Of the 18,974 participants surveyed at both baseline and the two-year follow-up, 6732 were diagnosed with MetS at baseline. **Table 1** outlines the demographic and clinical characteristics of these individuals. Notably, those with MetS had a significantly higher median CVAI compared to their non-MetS counterparts. **Supplemental Table 1** presents the characteristics of all study participants stratified by CVAI quartile. The median CVAI for all study participants was 90.49 (IQR, 68.66–114.58).

**Table 1:** Baseline characteristics of all participants.

Variable	Non-MetS (N=12242, 64.5%)	MetS (N=6732, 35.5%)	p value
Individual characteristics			
**Males, N (%)**	5176 (42.3)	3168 (47.1)	<0.0001 ^∗^
**Age, years**	56 (49-66)	61 (53-69)	<0.0001 ^∗^
**Education, N (%)**			<0.0001 ^∗^
Primary school or below	4209 (34.4)	2667 (39.6)	
Middle school	4541 (37.1)	2311 (34.3)	
High school or above	3492 (28.5)	1754 (26.1)	
**Annual Income, N (%)**			0.002 ^∗^
<5000 CNY	3097 (25.3)	1770 (26.3)	
5000-9999 CNY	1379 (11.3)	832 (12.4)	
10000-19999 CNY	1612 (13.2)	935 (13.9)	
≥20000 CNY	6154 (50.3)	3195 (47.4)	
Medical history and risk factor, N (%)			
Current smoking	2435 (19.9)	1567 (23.3)	<0.0001 ^∗^
Alcohol consumption	1871 (15.3)	1033 (15.3)	0.911
Physical inactivity	3083 (25.2)	1867 (27.7)	<0.0001 ^∗^
Hypertension	2533 (20.7)	4341 (64.5)	<0.0001 ^∗^
Diabetes	1251 (10.2)	2861 (42.5)	<0.0001 ^∗^
Dyslipidemia	2528 (20.7)	4410 (65.5)	<0.0001 ^∗^
Prior stroke	293 (2.4)	390 (5.8)	<0.0001 ^∗^
Family history of stroke	1101 (9.0)	803 (11.9)	<0.0001 ^∗^
General observation indexes			
SBP, mmHg	123 (115-132)	138 (130-150)	<0.0001 ^∗^
DBP, mmHg	77(70-82)	82 (76-90)	<0.0001 ^∗^
FBG, mmol/L	5.02 (4.52-5.55)	5.90 (4.99-7.59)	<0.0001 ^∗^
HbA1c, %	5.30 (5.00-5.70)	5.60 (5.10-6.70)	<0.0001 ^∗^
TG, mmol/L	1.25 (0.98-1.55)	2.05 (1.58-2.75)	<0.0001 ^∗^
TC, mmol/L	4.62 (3.98-5.30)	4.96 (4.20-5.67)	<0.0001 ^∗^
LDL-C, mmol/L	2.56 (2.09-3.11)	2.71 (2.10-3.29)	<0.0001 ^∗^
HDL-C, mmol/L	1.37 (1.17-1.65)	1.16 (0.97-1.43)	<0.0001 ^∗^
Indicators of adiposity			
BMI, kg/m^2^	23.18 (21.39-24.98)	24.54 (22.58-26.64)	<0.0001 ^∗^
WC, cm	80.0 (76.0-86.0)	84.0 (79.0-90.0)	<0.0001 ^∗^
CVAI	80.89 (62.09-101.08)	110.79 (88.45-133.32)	<0.0001 ^∗^

MetS, metabolic syndrome; SBP, systolic blood pressure; DBP, diastolic blood pressure; FBG, fasting blood glucose; HbA1C, glycosylated hemoglobin A 1c; TG, triglyceride; TC, total cholesterol; LDL-C, low-density lipoprotein cholesterol, HDL-C, high-density lipoprotein cholesterol; BMI, Body Mass Index, WC, waist circumferences, CVAI, Chinese visceral adiposity index. Statistical significance is considered at ^∗^ P < 0.05.

### 3.2 Association between CVAI and MetS at baseline

**Figure 1:**
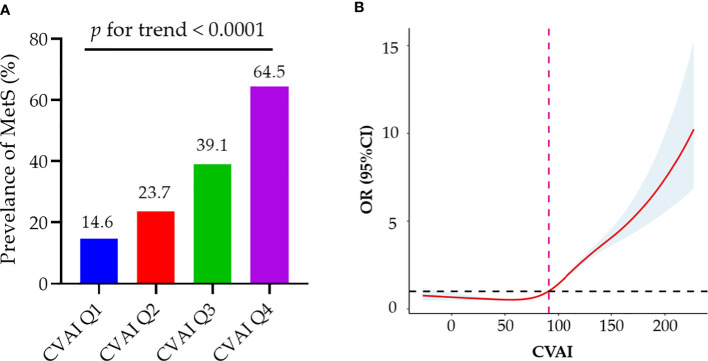
Associations of baseline CVAI with MetS. **(A)** As the CVAI increased, there was a corresponding rise in the prevalence of MetS; **(B)** Dose–response relationship of CVAI and MetS risk. CVAI could increase the risk of MetS when higher than 90.94. MetS, metabolic syndrome; CVAI, Chinese Visceral Adiposity Index; OR, odds ratio; CI, confidence interval.

As the CVAI increased, there was a corresponding increase in the prevalence of MetS (**Figure 1A**). **Table 2** shows a correlation between CVAI and MetS. In the unadjusted model, for each additional SD increase in CVAI, the risk of MetS increased by 1.59. In all two adjusted models, CVAI demonstrated an independent association with MetS; the adjusted odds ratios are 2.68 (95% CI: 2.57-2.79), and 2.05 (95% CI: 1.95-2.16), respectively. When assessed as quartiles, CVAI was significantly associated with MetS in the second, third, and fourth quartiles—even after adjustment for all confounding factors (adjusted OR: 1.54, 95% CI: 1.34, 1.76; adjusted OR: 2.83, 95% CI: 2.48, 3.24; adjusted OR: 6.29, 95% CI: 5.47,7.23, respectively). Participants in the third and fourth CVAI quartiles were associated with a significantly higher risk of MetS compared to their counterparts in the first and second quartiles (adjusted OR: 3.13, 95% CI: 2.86, 3.44). Additionally, dose-response relationships between CVAI and MetS were evaluated by restricted cubic splines (**Figure 1B**). CVAI increases the risk of MetS when higher than 90.94.

**Table 2:** Associations of baseline CVAI with MetS.

Variants	No. of case (%)	Crude		Model 1 ^a^		Model 2 ^b^	
		OR (95% CI)	p Value	OR (95% CI)	p Value	OR (95% CI)	p Value
**Per SD increase**		2.59 (2.49-2.69)	<0.0001^∗^	2.68 (2.57-2.79)	<0.0001^∗^	2.05 (1.95-2.16)	<0.0001^∗^
Quartiles							
Quartile 1 (< 68.66)	694/4743 (14.6)	Reference		Reference		Reference	
Quartile 2 (≥ 68.66 & < 90.49)	1126/4745 (23.7)	1.82 (1.64-2.02)	<0.0001^∗^	1.88 (1.69-2.09)	<0.0001^∗^	1.54 (1.34-1.76)	<0.0001^∗^
Quartile 3 (≥ 90.49 & < 114.58)	1852/4741 (39.1)	3.74 (3.39-4.13)	<0.0001^∗^	3.99 (3.60-4.43)	<0.0001^∗^	2.83 (2.48-3.24)	<0.0001^∗^
Quartile 4 (≥ 114.58)	3060/4745 (64.5)	10.60 (9.59-11.71)	<0.0001^∗^	11.63 (10.44-12.96)	<0.0001^∗^	6.29 (5.47-7.23)	<0.0001^∗^
*p* for trend			<0.0001^∗^		<0.0001^∗^		<0.0001^∗^
Categories							
Quartile 1-2 (< 90.49)	1820/9488 (19.2)	Reference		Reference		Reference	
Quartile 3-4 (≥ 90.49)	4912/9486 (51.8)	4.52 (4.24-4.83)	<0.0001^∗^	4.49 (4.19-4.82)	<0.0001^∗^	3.13 (2.86-3.44)	<0.0001^∗^

CVAI, Chinese visceral adiposity index; MetS, metabolic syndrome; OR, odds ratio; CI, confidence interval, SD, standard deviation.

^a^ Model 1 contained individual characteristics (age, sex, education, economic status). ^b^ Model 2 added lifestyle risk factors and medical history (smoking, alcohol drinking and physical activity, hypertension, diabetes mellitus, dyslipidemia, prior stroke, family medical history of stroke on the base of model 1. Statistical significance is considered at ^∗^ P < 0.05.

**Figure 2:**
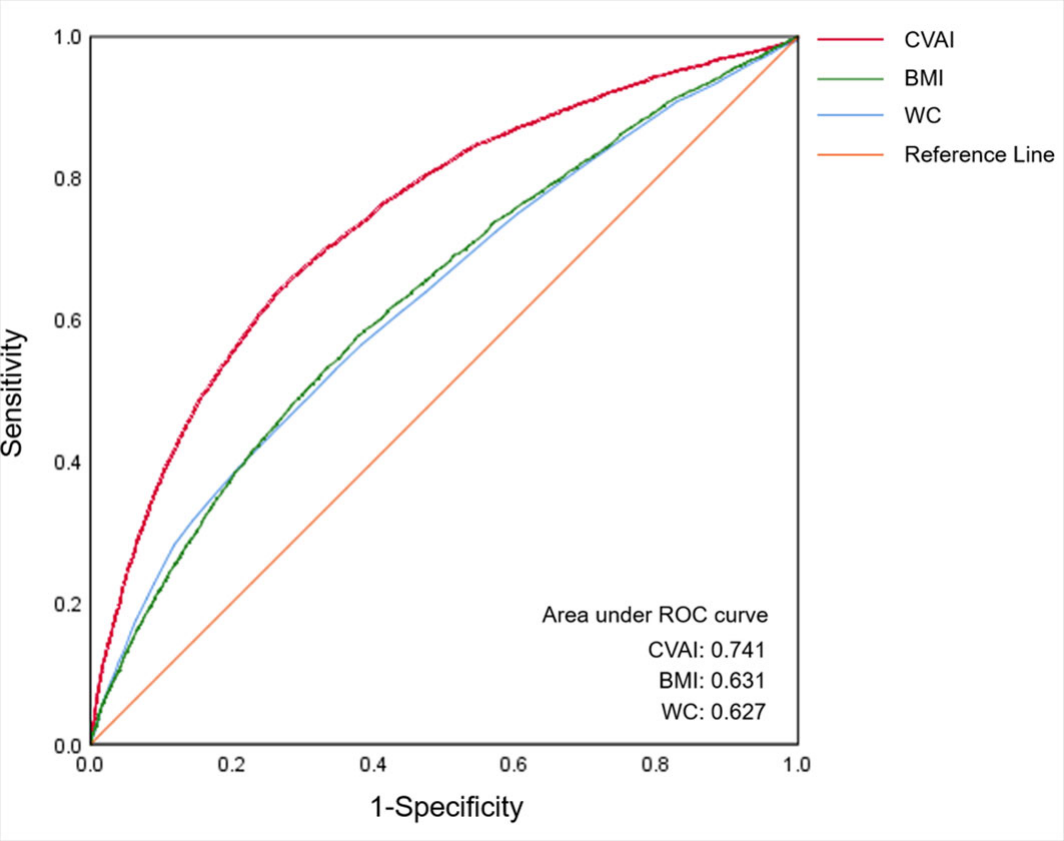
The ROC curves of CVAI, BMI and WC for MetS diagnosis. The area under the ROC curve of CVAI was 0.741 for diagnosing MetS, which is significantly superior to BMI and WC among Chinese adults (all p < 0.05). ROC, receiver-operating characteristic; CVAI, Chinese visceral adiposity index; BMI, body mass index; WC, waist circumference.

ROC curve analysis was used to compare the diagnostic efficacy of CVAI with that of BMI and WC in detecting MetS (**Figure 2**). In the diagnosis of MetS, the CVAI demonstrated the highest AUC values (AUC: 0.741, 95% CI: 0.734-0.749), exceeding those of WC (AUC: 0.627, 95% CI: 0.619-0.635) and BMI (AUC: 0.631, 95% CI: 0.623-0.639). **Table 3** presents the diagnostic performance of each anthropometric index in identifying MetS, encompassing sensitivity, specificity, and corresponding optimal cut-off values. CVAI exhibited the highest Youden indices (0.376) for identifying MetS, with an optimal cut-off of 99.15.

**Table 3:** ROC for BMI, WC, and CVAI in predicting MetS and cut-off points.

Parameter	BMI	WC	CVAI
Area under ROC curve (95% CI)	0.631 (0.623-0.639)	0.627 (0.619-0.635)	0.741 (0.734-0.749)
*p* value	<0.0001^∗^	<0.0001^∗^	<0.0001^∗^
Cut-off point	83.25	23.98	99.15
Youden Index	0.184	0.200	0.376
Sensitivity	0.530	0.578	0.646
Specificity	0.654	0.622	0.730

ROC, receiver operating characteristic; BMI, body mass index; WC, waist circumference; CVAI, Chinese visceral adiposity index; MetS, metabolic syndrome; CI, confidence interval. Statistical significance is considered at ^∗^ P < 0.05.

### 3.3 Comparison of baseline characteristics between MetS patients with and without stroke

Of the 6,732 patients with MetS, 72 experienced strokes during the two-year follow-up period; these individuals were more likely to be of advanced age and have a lower income at baseline relative to those without a history of stroke. Hypertension, previous history of stroke, and family history of stroke were more prevalent among individuals with a stroke incident. Additionally, the stroke group exhibited significantly higher SBP levels, BMI, WC, and CVAI at baseline (**Supplement Table 2**).

**Table 4** shows the characteristics of participants with MetS categorized by CVAI quartile. The median CVAI was 110.79 (IQR, 88.45–133.32). Participants in the upper CVAI quartile tended to be older, female, less educated, and have a higher income. They were also less physically active, and hypertension, dyslipidemia, prior stroke history, and family history of stroke were more prevalent among them. Furthermore, we observed a significant trend towards increasing SBP, TG, and TC levels, as well as decreasing HDL-C levels with increasing CVAI, among the participants who developed MetS.

**Table 4:** Baseline characteristics of MetS patients divided by CVAI quartiles.

Variants	CVAI Q1	CVAI Q2	CVAI Q3	CVAI Q4	p for trend
**Range**	<88.45	≥ 88.45 & < 110.79	≥ 110.79 & < 133.32	≥133.32	
**N**	1683	1683	1683	1683	
Individual characteristics					
**Males, N (%)**	995 (59.1)	714 (42.4)	664 (39.5)	795 (47.2)	<0.0001^∗^
**Age, years**	55 (49-64)	59 (53-67)	63 (55-69)	66 (57-72)	<0.0001^∗^
**Education, N (%)**					<0.0001^∗^
Primary school or below	596 (35.4)	642 (38.1)	688 (40.9)	741 (44.0)	
Middle school	633 (37.6)	593 (35.2)	555 (33.0)	530 (31.5)	
High school or above	454 (27.0)	448 (26.6)	440 (26.1)	412 (24.5)	
**Annual Income, N (%)**					0.003^∗^
<5000 CNY	450 (26.7)	459 (27.3)	432 (25.7)	429 (25.5)	
5000-9999 CNY	245 (14.6)	219 (13.0)	186 (11.1)	182 (10.8)	
10000-19999 CNY	236 (14.0)	227 (13.5)	235 (14.0)	237 (14.1)	
≥20000 CNY	752 (44.7)	778 (46.2)	830 (49.3)	835 (49.6)	
Medical history and risk factor, N (%)					
Current smoking	446 (26.5)	352 (20.9)	339 (20.1)	430 (25.5)	0.431
Alcohol consumption	268 (15.9)	220 (13.1)	242 (14.4)	303 (18.0)	0.055
Physical inactivity	424 (25.2)	424 (25.2)	454 (27.0)	565 (33.6)	<0.0001^∗^
Hypertension	966 (57.4)	1035 (61.5)	1147 (68.2)	1193 (70.9)	<0.0001^∗^
Diabetes	750 (44.6)	711 (42.2)	687 (40.8)	713 (42.4)	0.137
Dyslipidemia	925 (55.0)	1047 (62.2)	1152 (68.4)	1286 (76.4)	<0.0001^∗^
Prior atrial fibrillation	16 (1.0)	12 (0.7)	20 (1.2)	20 (1.2)	0.267
Prior stroke	71 (4.2)	96 (5.7)	96 (5.7)	127 (7.5)	<0.0001^∗^
Family history of stroke	166 (9.9)	197 (11.7)	221 (13.1)	219 (13.0)	0.002^∗^
General observation indexes					
SBP, mmHg	136 (130-148)	137 (130-149)	139 (130-152)	140 (130-154)	<0.0001^∗^
DBP, mmHg	82 (76-90)	82 (76-90)	83 (77-90)	83 (76-90)	0.290
FBG, mmol/L	5.99 (5.00-7.80)	5.80 (4.90-7.70)	5.70 (4.90-7.25)	5.99 (5.04-7.59)	0.790
HbA1c, %	5.60 (5.10-6.80)	5.70 (5.20-6.60)	5.60 (5.10-6.50)	5.70 (5.20-6.70)	0.096
TG, mmol/L	1.80 (1.22-2.25)	1.99 (1.54-2.50)	2.11 (1.70-2.91)	2.34 (1.79-3.37)	<0.0001^∗^
TC, mmol/L	4.89 (4.07-5.59)	5.01 (4.25-5.72)	4.96 (4.25-5.66)	4.97 (4.21-5.71)	0.010^∗^
LDL-C, mmol/L	2.60 (2.06-3.20)	2.77 (2.14-3.35)	2.75 (2.13-3.34)	2.70 (2.10-3.29)	0.068
HDL-C, mmol/L	1.30 (1.01-1.69)	1.20 (0.99-1.46)	1.13 (0.96-1.37)	1.06 (0.90-1.29)	<0.0001^∗^
Indicators of adiposity					
BMI, kg/m^2^	22.43 (20.95-23.87)	23.82 (22.22-25.33)	25.10 (23.44-26.87)	27.33 (25.51-29.22)	<0.0001^∗^
WC, cm	78.0 (73.0-80.0)	82.0 (78.0-85.0)	87.0 (83.0-90.0)	94.0 (89.0-99.0)	<0.0001^∗^

MetS, metabolic syndrome; CVAI, Chinese visceral adiposity index; SBP, systolic blood pressure; DBP, diastolic blood pressure; FBG, fasting blood glucose; HbA1C, glycosylated hemoglobin A 1c; TG, triglyceride; TC, total cholesterol; LDL-C, low-density lipoprotein cholesterol, HDL-C, high-density lipoprotein cholesterol; BMI, Body Mass Index, WC, waist circumferences. Statistical significance is considered at ^∗^ P < 0.05.

### 3.4 Association between CVAI at baseline and incident stroke during the 2-year follow-up of MetS patients

**Figure 3:**
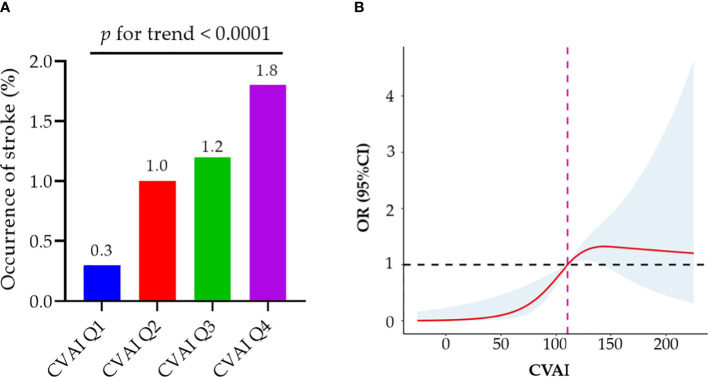
Associations of baseline CVAI with incident stroke among MetS patients. **(A)** MetS patients in the higher quartile of CVAI demonstrate a significantly elevated risk of stroke; **(B)** Dose–response relationship of CVAI and stroke risk. A reduced stroke risk was observed when the CVAI was either less than 110.91. MetS, metabolic syndrome; CVAI, Chinese Visceral Adiposity Index; OR, odds ratio; CI, confidence interval.

There was a positive association between CVAI and the risk of incident stroke (**Figure 3A**). **Table 5** presents the association between CVAI and incident stroke. In the unadjusted model, the risk of incident stroke increased by 64% (95% CI: 1.30, 2.06) for each SD of CVAI. In both adjusted model 1 and model 2, there was a significant association between the increase in CVAI and the occurrence of stroke, as evidenced by the adjusted ORs of 1.61 (95% CI: 1.26, 2.04) and 1.52 (95% CI: 1.18, 1.95), respectively. When assessing CVAI as quartiles, the associations between incident stroke and the second, third, and fourth CVAI quartiles were 3.22 (95% CI: 1.17-8.81), 4.04 (95% CI: 1.51-10.78), and 6.30 (95% CI: 2.44-16.24), respectively, relative to the first CVAI quartile. These association estimators did not change significantly after additional adjustment for individual characteristics, medical history, and risk factors. Consistently, participants in the third and fourth CVAI quartiles showed a significantly higher risk of incident stroke than those in the first and second quartiles (adjusted OR: 2.14, 95% CI: 1.25, 3.69).

**Table 5:** Associations of baseline CVAI with incident stroke in MetS patients.

Variants	No. of case (%)	Crude		Model 1 ^a^		Model 2 ^b^	
		OR (95% CI)	p Value	OR (95% CI)	p Value	OR (95% CI)	p Value
**Per SD increase**		1.64 (1.30-2.06)	<0.0001^∗^	1.61 (1.26-2.04)	<0.0001^∗^	1.52 (1.18-1.95)	<0.0001^∗^
Quartiles							
Quartile 1 (< 88.45)	5/1683 (0.3)	Reference		Reference		Reference	
Quartile 2 (≥ 88.45 & < 110.79)	16/1683 (1.0)	3.22 (1.17-8.81)	0.023^∗^	3.25 (1.18-8.96)	0.023^∗^	3.08 (1.11-8.52)	0.030^∗^
Quartile 3 (≥ 110.79 & < 133.32)	20/1683 (1.2)	4.04 (1.51-10.78)	0.005^∗^	4.12 (1.52-11.17)	0.005^∗^	3.78 (1.38-10.32)	0.010^∗^
Quartile 4 (≥133.32)	31/1683 (1.8)	6.30 (2.44-16.24)	<0.0001^∗^	6.03 (2.29-15.90)	<0.0001^∗^	5.34 (1.99-14.32)	0.001^∗^
*p* for trend			<0.0001^∗^		<0.0001^∗^		0.001^∗^
Categories							
Quartile 1-2 (< 110.79)	21/3366 (0.6)	Reference		Reference		Reference	
Quartile 3-4 (≥ 110.79)	51/3366 (1.5)	2.45 (1.47-4.08)	0.001^∗^	2.35 (1.38-3.98)	0.002^∗^	2.14 (1.25-3.69)	0.006^∗^

CVAI, Chinese visceral adiposity index; MetS, metabolic syndrome; OR, odds ratio; CI, confidence interval; SD, standard deviation.

^a^ Model 1 contained individual characteristics (age, sex, education, economic status). ^b^ Model 2 added lifestyle risk factors and medical history (smoking, alcohol drinking and physical activity, hypertension, diabetes mellitus, dyslipidemia, atrial fibrillation, prior stroke, family medical history of stroke on the base of model 1. Statistical significance is considered at ^∗^ P < 0.05.

As demonstrated by the logistic regression models, our findings also indicate a positive correlation between WC and incident stroke in individuals with MetS. Participants exhibiting abdominal obesity had a relative risk of 2.73 (95% CI: 1.67, 4.46) for developing an incident stroke compared to their counterparts without abdominal obesity (**Supplement Table 3**). The fully adjusted model revealed significant associations between incident stroke and the third and fourth BMI quartiles in patients with MetS, but not the second quartile (**Supplement Table 4**). Although general obesity may be as-sociated with an elevated risk of stroke, the association did not reach statistical significance after adjustment for confounding factors (adjusted OR: 1.60; 95% CI: 0.99, 2.59). We further used restricted cubic splines to visualize the relation of CVAI, WC, and BMI with incident stroke in patients with MetS. The risk of incident stroke was non-linearly associated with CVAI (**Figure 3B**) and followed an inverted U-shaped curve with respect to BMI (**Supplement Figure 2A**). A decreased risk of stroke was observed when the CVAI was below 110.91.

During the two-year follow-up period, 57 strokes occurred among the 12,242 patients without MetS. However, no significant correlation was detected between CVAI and stroke in non-MetS patients (**Supplement Table 5**).

### 3.5 Subgroup analyses of stroke risk factors

To evaluate any potential modifying effects of stroke risk factors on the association between CVAI (3-4 quartiles vs. 1-2 quartiles) and incident stroke, subgroup analyses were conducted (**Table 6**). We found no significant interaction between CVAI and covariates in relation to incident stroke.

**Table 6:** Subgroup analysis between CVAI with stroke.

Characteristics	Crude OR (95%CI)	p for interaction	Adjusted OR (95%CI) ^a^	Adjusted p for interaction ^a^
**Age, years**		0.274		0.512
<60	2.18 (0.86-5.53)		1.97 (0.73-5.33)	
≥60	2.03 (1.08-3.80)		2.13 (1.10-4.13)	
**Sex**		0.703		0.384
Male	2.72 (1.29-5.74)		2.49 (1.15-5.41)	
Female	2.23 (1.11-4.49)		1.73 (0.79-3.76)	
**Diabetes**		0.846		0.836
Yes	2.59 (1.28-5.25)		2.27 (1.06-4.87)	
No	2.34 (1.12-4.91)		2.13 (0.98-4.65)	
**Hypertension**		0.341		0.377
Yes	2.01 (1.16-3.51)		1.95 (1.08-3.53)	
No	4.02 (1.09-14.88)		3.31 (0.82-13.29)	
**Dyslipidemia**		0.230		0.319
Yes	3.19 (1.59-6.41)		2.73 (1.32-5.64)	
No	1.65 (0.72-3.75)		1.46 (0.60-3.57)	
**Prior stroke**		0.386		0.464
Yes	1.52 (0.51-4.54)		2.27 (0.64-8.13)	
No	2.63 (1.47-4.70)		2.25 (1.22-4.16)	
**Current smoking**		0.375		0.224
Yes	3.59 (1.32-9.77)		3.63 (1.28-10.27)	
No	2.12 (1.17-3.84)		1.65 (0.87-3.16)	
**Alcohol consumption**		0.482		0.558
Yes	4.08 (0.88-18.98)		2.91 (0.55-15.28)	
No	2.27 (1.32-3.92)		2.10 (1.17-3.75)	
**Physical inactivity**		0.763		0.636
Yes	2.16 (0.90-5.20)		2.01 (0.81-5.03)	
No	2.55 (1.36-4.78)		2.25 (1.15-4.42)	

CVAI, Chinese visceral adiposity index; OR, odds ratio; CI, confidence interval.

^a^ Adjusted for all covariates except effect modifier. Statistical significance is considered at ^∗^ P < 0.05.

## 4 Discussion

The present study confirms the superiority of CVAI over BMI and WC in identifying individuals with MetS in a large Chinese population. Furthermore, this investigation is, to the best of our knowledge, the first to detect a robust positive correlation between incident stroke and CVAI in patients with MetS. Our findings suggest a strong association between elevated levels of CVAI and incident stroke in Chinese adults diagnosed with MetS and that reducing CVAI levels could help to mitigate the risk of stroke in patients with MetS.

Epidemiologic studies have demonstrated that the standardized prevalence of MetS is 31.1% among Chinese individuals aged ≥20 years (37). Furthermore, as the obese population continues to increase, so too does the incidence of MetS. The baseline prevalence of MetS in our sample population (35.5%) was consistent with statistics reported in previous epidemiological research (37). The excessive accumulation of abdominal visceral fat is characteristic of MetS and clinically significant when assessing MetS in individuals of normal body weight (18). In the Chinese population, CVAI has been proposed as a valid, dependable indicator of visceral adiposity dysfunction (38). Our findings indicate that CVAI levels are significantly higher among those with MetS than those without it. Although previous studies have shown that CVAI has a favorable predictive performance for detecting MetS (39, 40), their findings were limited by having been derived from relatively small sample sizes. By contrast, our investigation demonstrates a significant positive association between CVAI levels and MetS in a large study population. CVAI exhibited superior performance in the diagnosis of MetS among Chinese adults, as evidenced by a higher AUC and greater overall discriminative ability than those of BMI and WC. This result is consistent with the findings of the previous research (39, 40), suggesting that CVAI, a quantitative index of visceral fat, can serve as a more reliable indicator of MetS than either BMI or WC.

The present study has also revealed that MetS patients in the higher quartile of CVAI were associated with lower levels of educational attainment. This finding may be attributed to the fact that individuals with higher levels of education are more inclined towards adopting healthier behaviors, which in turn may lead to a greater likelihood of remission from MetS (41). Moreover, it was noted that individuals diagnosed with MetS displayed a significant positive association between their income level and CVAI, signifying a rise in visceral adiposity with an increase in income (42–44). The observed trend is incongruent with the findings in developed nations, where individuals with lower incomes typically exhibit elevated rates of obesity and MetS. One plausible explanation for this disparity may be linked to the inclination of individuals with slightly higher incomes in developing countries to consume highly processed foods that lack nutritional value and contain “empty calories,” which are additional to their daily diet (44). Future studies ought to explore the correlation between visceral adiposity and dietary patterns within developing country populations.

Patients diagnosed with MetS are considered at high-risk for stroke (8–10). The current study demonstrates that the incidence of stroke during 2-year follow-up in patients with MetS is 2.31 times higher than it is for their counterparts without MetS, underscoring the importance of preventing stroke incidence in MetS patients. Mendelian randomization studies have suggested that visceral adiposity exerts a more potent impact on stroke risk than does general adiposity (45). Furthermore, visceral adiposity has been causally linked to the occurrence of stroke (46). While research on the longitudinal associations between visceral adiposity and stroke risk in patients with MetS has been limited, the present large population-based prospective cohort study found a strong association between elevated CVAI and incident stroke in Chinese adults who experienced MetS—even after adjustment for potential confounders. Specifically, each 1 SD increase in CVAI was found to increase the risk of incident stroke by a factor of 1.52 after correction. These findings provide additional support for the potential of reducing CVAI as a strategy to lower the risk of incident stroke among individuals with MetS.

Previous research has indicated that individuals with MetS and a BMI indicative of general obesity are at an increased risk of developing CVD relative to those without general obesity [17]. In our study, participants with general obesity were also found to have a higher risk of incident stroke relative to their non-obese counterparts; however, this trend did not reach statistical significance. The restricted cubic spline analysis revealed a dose-dependent association between CVAI and an increased risk of incident stroke. However, as BMI increases, an inverted U-shaped dose-response relationship was observed. One possible explanation is that BMI is unable to distinguish between the accumulation of fat-free mass and adipose tissue, leading to misidentification of individuals with high muscle mass as overweight or obese. The aforementioned data suggests that CVAI reflects stroke risk better than does BMI. Thus, in the routine practice of stroke prevention, assessing excessive body weight should incorporate an indicator of visceral adiposity rather than BMI alone.

Multiple factors may account for the correlation between CVAI and stroke in patients with MetS. First, CVAI levels are influenced by dyslipidemia, a common vascular risk factor for stroke associated with MetS. Second, the relationship might be explained by a demonstrated correlation between CVAI and the early development of traditional risk factors for stroke: e.g., Han et al. demonstrated a positive correlation between elevated CVAI and heightened risks of diabetes (47), a well-documented risk factor for stroke. The association between elevated CVAI and an increased risk of developing hypertension provides further support for the connection between CVAI and stroke risk factors (48). Finally, visceral adipose tissue not only secretes elevated levels of pro-inflammatory cytokines (49, 50), but also fulfills endocrine functions and plays a pivotal role in the pathogenesis of insulin resistance (51). The pathophysiological alterations prompted by visceral adipose tissue may exacerbate the risk of atherosclerosis, a major contributor to ischemic stroke. Indeed, enhanced levels of CVAI are positively associated with an increased likelihood of carotid atherosclerotic plaque formation (28, 52). Subsequent investigations could further explore the correlation between CVAI and serum concentrations of pro-inflammatory cytokines.

Interestingly, the present investigation did not observe any significant association between CVAI and stroke in non-MetS patients. This finding is consistent with those of Mirzaei et al., who observed no significant elevation in the risk of CVD in either metabolically healthy normal weight and metabolically healthy obese (MHO) groups after a 12-year follow-up period (53). A 14-year population-based prospective study of 5,314 individuals aged ≥55 years in Rotterdam also found that MHO did not augment the risk of cardiovascular disease (54). One possible explanation for this finding is that the incidence of cardiovascular events may be more closely linked to progression towards metabolic syndrome than obesity in middle-aged and older individuals (55). Further investigations are warranted to examine the contribution of CVAI in MetS risk among individuals with metabolically healthy normal weight and MHO.

This study is, to the best of our knowledge, the first study to investigate a correlation between CVAI and incident stroke in a robust sample size of Chinese MetS patients. Specifically, a gradual increase in the risk of stroke was observed when the CVAI surpassed the threshold of 110.91. Therefore, it is recommended that individuals diagnosed with MetS undergo further medical examinations to determine their susceptibility to stroke when their CVAI score exceeds 110.91 and promptly implement preventive measures against stroke. While this study benefitted from meticulous data collection and rigorous adjustment for confounding factors, it is subject to the following limitations. The 2-year follow-up period allowed for only a limited number of events for analysis. For which reason, the logistic regression models did not meet the criterion of 10 events per variable (EPV) in analyzing stroke risk among MetS patients. Nonetheless, it has been recommended that sample sizes of 5 to 10 EPV included in a regression equation could yield fairly stable coefficients in logistic regression models [44,45]. Moreover, our results exhibited a degree of resemblance to analogous previous investigations. Hence, it is suggested that our results are robust to some extent. Additionally, a 2-year follow-up period remains a reasonable timeframe for identifying individuals with an elevated risk of stroke. In addition to long-term risk information, short-term stroke risk information may also be of interest and more persuasive for behavior modification. An additional limitation of this study pertains to its geographical scope, as it was solely carried out in Hunan Province. Therefore, further validation across other provinces or larger populations is necessary to substantiate the universality of the findings. Finally, the impact of diet and medication on incident stroke was not taken into account due to data limitations.

## 5 Conclusions

CVAI demonstrates an independent and positive correlation with MetS, outperforming BMI and WC in identifying individuals with MetS. These findings recommend CVAI as a superior screening tool for MetS. Furthermore, elevated levels of CVAI are strongly linked to incident stroke in Chinese adults diagnosed with MetS, suggesting that reducing CVAI levels can mitigate the risk of stroke in individuals with MetS.

## Data availability statement

The raw data supporting the conclusions of this article will be made available by the authors, without undue reservation.

## Ethics statement

The studies involving human participants were reviewed and approved by Xiangya Hospital Ethics Committee. The protocol and informed consent for the study of the China Stroke High-risk Population Screening and Intervention Program were reviewed and approved by the Institutional Review Board at the Capital Medical University Xuanwu Hospital early. The patients/participants provided their written informed consent to participate in this study.

## Author contributions

Conceptualization, JX and ZL. Methodology, QH, BD and ZL. Software, QH and XF. Validation, YD, FY and JF. Formal analysis, ZL and MW. Investigation, XF. Resources and data curation, JX. Writing—original draft preparation, ZL. Writing—review and editing, and project administration, JX. All authors have read and agreed to the published version of the manuscript.
