# Supplementary material for: Elevated Chinese visceral adiposity index increases the risk of stroke in Chinese patients with metabolic syndrome
Source: Front Endocrinol (Lausanne). 2023 Jun 29;14:1218905. doi: 10.3389/fendo.2023.1218905 (PMC10339806; doi:10.3389/fendo.2023.1218905)
Supplement: Supplementary file 1 [file DataSheet_1.docx]

Supplementary Material

Elevated Chinese visceral adiposity index increases the risk of stroke in Chinese patients with metabolic syndrome

**Zeyu Liu, Qin Huang, Bi Deng, Minping Wei, Xianjing Feng, Fang Yu, Jie Feng, Yang Du, Jian Xia ***

*** Correspondence:** Jian Xia: xjian1216@csu.edu.cn.

**Supplement Table 1.** Baseline characteristics of all participants divided by CVAI quartiles.

| **Variants** | **CVAI Q1** | **CVAI Q2** | **CVAI Q3** | **CVAI Q4** | ***p* for trend** |
| --- | --- | --- | --- | --- | --- |
| **Range** | <68.66 | ≥ 68.66 & <90.49 | ≥90.49 & < 114.58 | ≥ 114.58 |  |
| **N** | 4744 | 4744 | 4744 | 4744 |  |
| **Individual characteristics** | | | | | |
| **Males, N (%)** | 2230 (47.0) | 1995 (42.1) | 1983 (41.8) | 2136 (45.0) | 0.055 |
| **Age, years** | 51 (46-59) | 55 (50-64) | 61 (54-68) | 66 (57-72) | <0.0001^∗^ |
| **Education, N (%)** |  |  |  |  | <0.0001^∗^ |
| Primary school or below | 1369 (28.9) | 1593 (33.6) | 1867 (39.4) | 2047 (43.1) |  |
| Middle school | 1836 (38.7) | 1833 (38.6) | 1632 (34.4) | 1551 (32.7) |  |
| High school or above | 1539 (32.4) | 1318 (27.8) | 1242 (26.2) | 1147 (24.2) |  |
| **Annual Income, N (%)** |  |  |  |  | 0.042^∗^ |
| <5000 CNY | 1107 (23.3) | 1226 (25.8) | 1286 (27.1) | 1248 (26.3) |  |
| 5000-9999 CNY | 591 (12.5) | 576 (12.1) | 544 (11.5) | 500 (10.5) |  |
| 10000-19999 CNY | 630 (13.3) | 660 (13.9) | 632 (13.3) | 625 (13.2) |  |
| ≥20000 CNY | 2416 (50.9) | 2282 (48.1) | 2279 (48.1) | 2372 (50.0) |  |
| **Medical history and risk factor, N (%)** | | | | | |
| Current smoking | 1023 (21.6) | 899 (19.0) | 967 (20.4) | 1113 (23.5) | 0.007^∗^ |
| Alcohol consumption | 749 (15.8) | 649 (13.7) | 687 (14.5) | 819 (17.3) | 0.025^∗^ |
| Physical inactivity | 1203 (25.4) | 1149 (24.2) | 1176 (24.8) | 1422 (30.0) | <0.0001^∗^ |
| Hypertension | 949 (20.0) | 1384 (29.2) | 1859 (39.2) | 2682 (56.5) | <0.0001^∗^ |
| Diabetes | 734 (15.5) | 874 (18.4) | 1056 (22.3) | 1448 (30.5) | <0.0001^∗^ |
| Dyslipidemia | 985 (20.8) | 1370 (28.9) | 1894 (39.9) | 2689 (56.7) | <0.0001^∗^ |
| Prior stroke | 73 (1.5) | 125 (2.6) | 193 (4.1) | 292 (6.2) | <0.0001^∗^ |
| Family history of stroke | 378 (8.0) | 466 (9.8) | 493 (10.4) | 567 (11.9) | <0.0001^∗^ |
| **General observation indexes** | | | | | |
| SBP, mmHg | 123 (114-132) | 127 (118-137) | 130 (120-140) | 135 (124-150) | <0.0001^∗^ |
| DBP, mmHg | 77 (70-82) | 78 (72-84) | 80 (72-86) | 80 (74-88) | <0.0001^∗^ |
| FBG, mmol/L | 5.09 (4.51-5.61) | 5.11 (4.60-5.80) | 5.22 (4.70-6.10) | 5.50 (4.80-6.62) | <0.0001^∗^ |
| HbA1c, % | 5.30 (5.00-5.70) | 5.40 (5.00-5.80) | 5.40 (5.00-5.90) | 5.60 (5.10-6.20) | <0.0001^∗^ |
| TG, mmol/L | 1.20 (0.91-1.57) | 1.31 (1.03-1.72) | 1.50 (1.15-2.10) | 1.88 (1.33-2.72) | <0.0001^∗^ |
| TC, mmol/L | 4.60 (3.94-5.27) | 4.67 (4.02-5.40) | 4.80 (4.10-5.52) | 4.84 (4.15-5.56) | <0.0001^∗^ |
| LDL-C, mmol/L | 2.48 (2.03-3.05) | 2.58 (2.10-3.11) | 2.66 (2.12-3.24) | 2.70 (2.11-3.29) | <0.0001^∗^ |
| HDL-C, mmol/L | 1.48 (1.23-1.83) | 1.34 (1.13-1.60) | 1.26 (1.07-1.51) | 1.17 (0.98-1.38) | <0.0001^∗^ |
| **Indicators of adiposity** | | | | | |
| BMI, kg/m^2^ | 21.60  (20.20-22.99) | 23.15  (21.67-24.51) | 24.09  (22.60-25.71) | 26.17  (24.44-28.04) | <0.0001^∗^ |
| WC, cm | 75.0 (70.0-78.0) | 80.0 (77.0-83.0) | 84.0 (80.0-87.0) | 90.0 (86.0-96.0) | <0.0001^∗^ |

**Abbreviations:** CVAI, Chinese visceral adiposity index; MetS, metabolic syndrome; SBP, systolic blood pressure; DBP, diastolic blood pressure; FBG, fasting blood glucose; HbA1C, glycosylated hemoglobin A 1c; TG, triglyceride; TC, total cholesterol; LDL-C, low-density lipoprotein cholesterol, HDL-C, high-density lipoprotein cholesterol; BMI, Body Mass Index, WC, waist circumferences. Statistical significance is considered at ^∗^ P < 0.05.

**Supplement Table 2.** Baseline characteristics of MetS patients divided by stroke outcome.

| **Variable** | **Non-stroke (N=6660)** | **Stroke (N=72)** | ***p* value** |
| --- | --- | --- | --- |
| **Individual characteristics** | | | |
| **Males, N (%)** | 3135 (47.1) | 33 (45.8) | 0.834 |
| **Age, years** | 61 (53-69) | 66 (59-72) | <0.0001^∗^ |
| **Education, N (%)** |  |  | 0.192 |
| Primary school or below | 2631 (39.5) | 36 (50.0) |  |
| Middle school | 2291 (34.4) | 20 (27.8) |  |
| High school or above | 1738 (26.1) | 16 (22.2) |  |
| **Annual Income, N (%)** |  |  | 0.002^∗^ |
| <5000 CNY | 1741 (26.1) | 29 (40.3) |  |
| 5000-9999 CNY | 818 (12.3) | 14 (19.4) |  |
| 10000-19999 CNY | 926 (13.9) | 9 (12.5) |  |
| ≥20000 CNY | 3175 (47.7) | 20 (27.8) |  |
| **Medical history and risk factor, N (%)** | | | |
| Current smoking | 1545 (23.2) | 22 (30.6) | 0.142 |
| Alcohol consumption | 1022 (15.3) | 11 (15.3) | 0.987 |
| Physical inactivity | 1842 (27.7) | 25 (34.7) | 0.183 |
| Hypertension | 4281 (64.3) | 60 (83.3) | 0.001^∗^ |
| Diabetes | 2823 (42.4) | 38 (52.8) | 0.076 |
| Dyslipidemia | 4361 (65.5) | 49 (68.1) | 0.648 |
| Prior atrial fibrillation | 68 (1.0) | 0 (0) | 1.000 |
| Prior stroke | 375 (5.6) | 15 (20.8) | <0.0001^∗^ |
| Family history of stroke | 786 (11.8) | 17 (23.6) | 0.002^∗^ |
| **General observation indexes** | | | |
| SBP, mmHg | 138 (130-150) | 148 (135-162) | <0.0001^∗^ |
| DBP, mmHg | 82(76-90) | 84 (77-90) | 0.756 |
| FBG, mmol/L | 5.89 (4.98-7.57) | 6.20 (5.29-7.80) | 0.188 |
| HbA1c, % | 5.60 (5.10-6.70） | 5.65 (5.23-6.68) | 0.767 |
| TG, mmol/L | 2.05 (1.58-2.75) | 2.18 (1.50-3.14) | 0.277 |
| TC, mmol/L | 4.96 (4.20-5.66) | 5.05 (4.27-5.86) | 0.485 |
| LDL-C, mmol/L | 2.71 (2.10-3.29) | 2.73 (2.19-3.47) | 0.420 |
| HDL-C, mmol/L | 1.16 (0.97-1.43) | 1.15 (0.98-1.45) | 0.987 |
| **Indicators of adiposity** | | | |
| BMI, kg/m2 | 24.52 (22.58-26.64) | 25.25 (23.55-27.16) | 0.021^∗^ |
| WC, cm | 84.0 (78.0-90.0) | 89.0 (83.0-93.0) | <0.0001^∗^ |
| CVAI | 110.54 (88.24-133.13) | 128.57 (107.72-147.27) | <0.0001^∗^ |

**Abbreviations:** MetS, metabolic syndrome; SBP, systolic blood pressure; DBP, diastolic blood pressure; FBG, fasting blood glucose; HbA1C, glycosylated hemoglobin A 1c; TG, triglyceride; TC, total cholesterol; LDL-C, low-density lipoprotein cholesterol, HDL-C, high-density lipoprotein cholesterol; BMI, Body Mass Index; WC, waist circumferences, CVAI, Chinese visceral adiposity index. Statistical significance is considered at ^∗^ P < 0.05.

**Supplement Table3.** Associations of baseline WC with stroke risk among MetS patients.

| **Variants** | **No. of case (%)** | **Crude** | | **Model 1 ^a^** | | **Model 2 ^b^** | |
| --- | --- | --- | --- | --- | --- | --- | --- |
|  |  | **OR (95% CI)** | ***p* Value** | **OR (95% CI)** | ***p* Value** | **OR (95% CI)** | ***p* Value** |
| **Per SD increase** |  | 1.48  (1.19-1.83) | <0.0001^∗^ | 1.58  (1.27-1.96) | <0.0001^∗^ | 1.49  (1.20-1.87) | <0.0001^∗^ |
| **Quartiles** | | | | | | | |
| Quartile 1  (< 79.0) | 10/1680 (0.6) | Reference |  | Reference |  | Reference |  |
| Quartile 2  (≥ 79.0& < 84.0) | 9/1488  (0.6) | 1.02  (0.41-2.51) | 0.972 | 1.10  (0.45-2.73) | 0.833 | 1.03  (0.42-2.56) | 0.946 |
| Quartile 31  (≥ 84.0 & < 90.0) | 19/1676 (1.1) | 1.92  (0.89-4.13) | 0.098 | 2.25  (1.03-4.87) | 0.041^∗^ | 2.02  (0.93-4.41) | 0.077 |
| Quartile 4  (≥ 90.0) | 34/1888 (1.8) | 3.06  (1.51-6.22) | 0.002^∗^ | 3.85  (1.87-7.94) | <0.0001^∗^ | 3.36  (1.62-7.00) | 0.001^∗^ |
| *p* for trend |  |  | <0.0001^∗^ |  | <0.0001^∗^ |  | <0.0001^∗^ |
| **Abdominal obesity** ^c^ | | | | | | | |
| No | 27/4144 (0.7) |  |  |  |  |  |  |
| Yes | 45/2588 (1.7) | 2.70  (1.67-4.36) | 0.001^∗^ | 2.98  (1.84-4.85) | <0.0001^∗^ | 2.73  (1.67-4.46) | <0.0001^∗^ |

**Abbreviations:** WC, waist circumferences; MetS, metabolic syndrome; OR, odds ratio; CI, confidence interval, SD, standard deviation.

^a^ Model 1 contained individual characteristics (age, sex, education, economic status). ^b^ Model 2 added lifestyle risk factors and medical history (smoking, alcohol drinking and physical activity, hypertension, diabetes mellitus, dyslipidemia, atrial fibrillation, prior stroke, family medical history of stroke on the base of model 1. ^c^ Abdominal obesity was defined as a WC of ≥90 cm for men and a WC of ≥85 cm for women. Statistical significance is considered at ^∗^ P < 0.05.

**Supplement Table 4.** Associations of baseline BMI with stroke risk among MetS patients.

| **Variants** | **No. of case (%)** | **Crude** | | **Model 1** ^a^ | | **Model 2** ^b^ | |
| --- | --- | --- | --- | --- | --- | --- | --- |
|  |  | **OR (95% CI)** | ***p* Value** | **OR (95% CI)** | ***p* Value** | **OR (95% CI)** | ***p* Value** |
| **Per SD increase** |  | 1.24  (0.99-1.54) | 0.054 | 1.34  (1.08-1.66) | 0.008 ^∗^ | 1.25  (1.00-1.57) | 0.046 ^∗^ |
| **Quartiles** | | | | | | | |
| Q1  (< 22.59) | 10/1687 (0.6) | Reference |  | Reference |  |  |  |
| Q2  (≥ 22.59 & < 24.54) | 15/1686 (0.9) | 1.51  (0.67-3.36) | 0.318 | 1.72  (0.77-3.86) | 0.187 | 1.66  (0.74-3.73) | 0.222 |
| Q3  (≥ 24.5 & < 26.62) | 25/1676 (1.5) | 2.54  (1.22-5.30) | 0.013 ^∗^ | 3.08  (1.46-6.46) | 0.003 ^∗^ | 2.84  (1.34-5.99) | 0.006 ^∗^ |
| Q4  (≥ 26.62) | 22/1683 (1.3) | 2.22  (1.05-4.71) | 0.037 ^∗^ | 2.83  (1.33-6.06) | 0.007 ^∗^ | 2.42  (1.12-5.25) | 0.025 ^∗^ |
| *p* for trend |  |  | 0.015 ^∗^ |  | 0.002 ^∗^ |  | 0.010 ^∗^ |
| **General obesity** ^c^ | | | | | | | |
| No | 33/3813 (0.9) | Reference |  | Reference |  | Reference |  |
| Yes | 39/2919 (1.3) | 1.55  (0.97-2.47) | 0.065 | 1.77  (1.11-2.84) | 0.018 ^∗^ | 1.60  (0.99-2.59) | 0.055 |

**Abbreviations:** BMI, Body Mass Index; MetS, metabolic syndrome; OR, odds ratio; CI, confidence interval, SD, standard deviation.

^a^ Model 1 contained individual characteristics (age, sex, education, economic status). ^b^ Model 2 added lifestyle risk factors and medical history (smoking, alcohol drinking and physical activity, hypertension, diabetes mellitus, dyslipidemia, atrial fibrillation, prior stroke, family medical history of stroke on the base of model 1. ^c^ General obesity was defined as BMI ≥ 25 kg/m^2^. Statistical significance is considered at ^∗^ P < 0.05.

**Supplement Table 5.** Associations of baseline CVAI with stroke risk among non-MetS participants.

| **Variants** | **No. of case (%)** | **Crude** | | **Model 1** ^a^ | | **Model 2** ^b^ | |
| --- | --- | --- | --- | --- | --- | --- | --- |
|  |  | **OR (95% CI)** | ***p* Value** | **OR (95% CI)** | ***p* Value** | **OR (95% CI)** | ***p* Value** |
| **Per SD increase** |  | 0.99  (0.77-1.29) | 0.960 | 0.95  (0.72-1.24) | 0.689 | 0.90  (0.68-1.18) | 0.434 |
| **Quartiles** | | | | | | | |
| Quartile 1  (< 62.09) | 9/3058  (0.3) | Reference |  | Reference |  | Reference |  |
| Quartile 2  (≥ 62.09 & < 80.89) | 21/3065  (0.7) | 2.34  (1.07-5.11) | 0.033^∗^ | 2.38  (1.08-5.24) | 0.031^∗^ | 2.29  (1.04-5.05) | 0.041^∗^ |
| Quartile 3  (≥ 80.89 & < 101.08) | 17/3058  (0.6) | 1.89  (0.84-4.26) | 0.122 | 1.82  (0.80-4.17) | 0.155 | 1.79  (0.78-4.13) | 0.171 |
| Quartile 4  (≥ 101.08) | 10/3061  (0.3) | 1.11  (0.45-2.74) | 0.820 | 0.94  (0.37-2.40) | 0.891 | 0.81  (0.31-2.11) | 0.662 |
| *p* for trend |  |  | 0.952 |  | 0.642 |  | 0.437 |

**Abbreviations:** CVAI, Chinese visceral adiposity index; MetS, metabolic syndrome; OR, odds ratio; CI, confidence interval, SD, standard deviation.

^a^ Model 1 contained individual characteristics (age, sex, education, economic status). ^b^ Model 2 added lifestyle risk factors and medical history (smoking, alcohol drinking and physical activity, hypertension, diabetes mellitus, dyslipidemia, atrial fibrillation, prior stroke, family medical history of stroke on the base of model 1. Statistical significance is considered at ^∗^ P < 0.05.


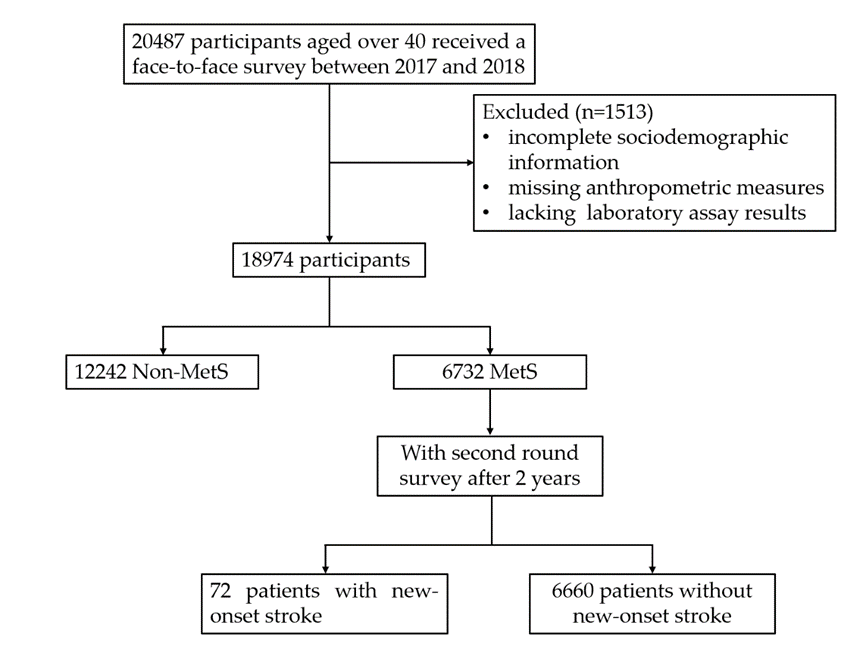


**Supplement Figure 1**. The process of study participants selection. MetS, metabolic syndrome.


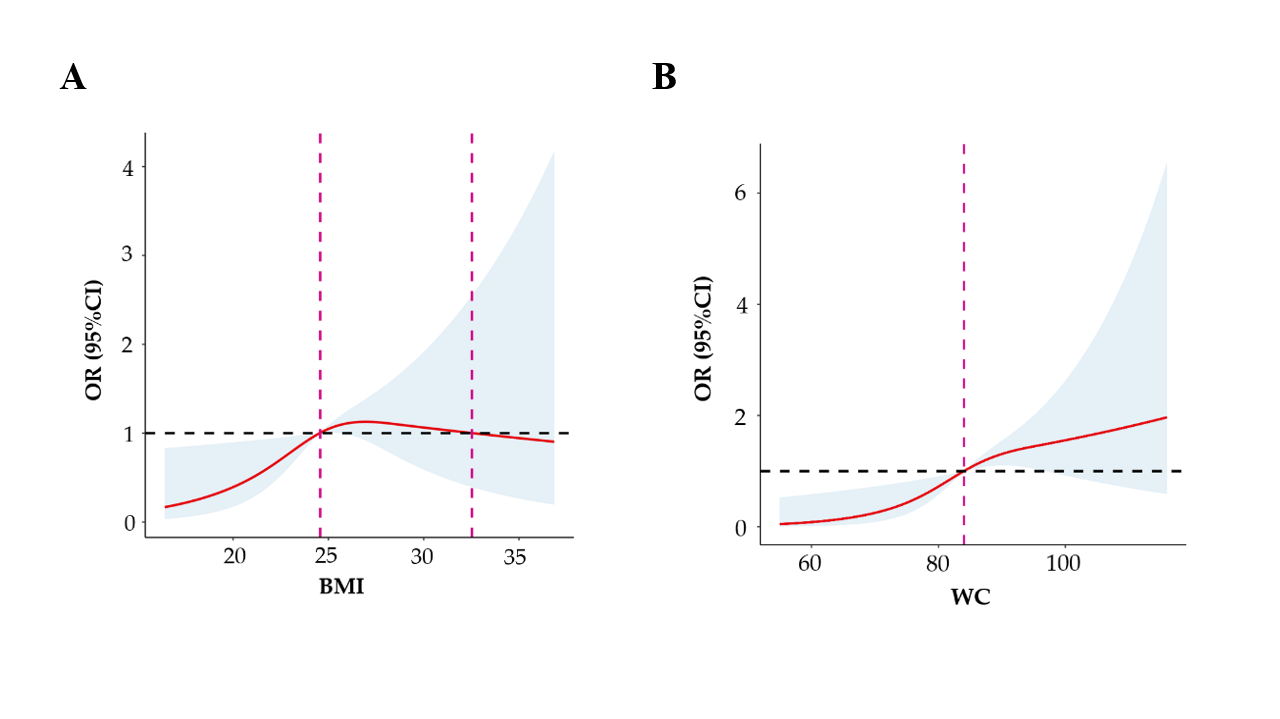


**Supplement Figure 2.** Dose–response relationship of BMI or WC and stroke risk among MetS patients. The risk of stroke increased in a linear manner with increasing waist circumference (b), while the relationship between BMI and stroke risk followed an inverted U-shaped curve (a). Abbreviations: MetS, metabolic syndrome; BMI, Body Mass Index; WC, waist circumferences OR, odds ratio; CI, confidence interval.
